# When normalization induces correlation: shared and unstable references can create hidden dependencies

**DOI:** 10.1038/s41540-026-00722-2

**Published:** 2026-04-21

**Authors:** Kiyoshi F. Fukutani, Thomas H. Hampton, Mattheus Luís Alves Santiago, Eddy José Francisco de Oliveira, Todd A. MacKenzie

**Affiliations:** 1 Universidade Estadual de Feira de Santana, Feira de Santana, Brazil; 2 Geisel School of Medicine, Dartmouth College, Hanover, NH USA; 3 The Dartmouth Institute for Health Policy and Clinical Practice, Dartmouth College, Hanover, NH USA

**Keywords:** Biological techniques, Computational biology and bioinformatics, Systems biology

## Abstract

Correlation analyses are widely used to infer biological relationships, yet preprocessing can introduce structural dependencies that inflate correlation estimates. Here, we examine an under-recognized mechanism: shared-reference transformations, in which multiple variables are centered or scaled using the same reference before correlation-based inference. We derive analytically that, even when two variables are independent, subtracting a shared reference induces nonzero covariance and yields an expected correlation that increases with the variance of the reference. Simulations show that this artifact is systematic, strengthens as the reference becomes more variable, and does not disappear with increasing sample size. We further show that sequential shared transformations of the form (X−Z)/W can become unstable when denominators fluctuate or approach zero, producing highly dispersed correlations consistent with heavy-tailed ratio effects. As an illustrative count-based example,we introduce a simulation with sample-level scaling showing that downstream shared-reference steps can reintroduce dependence after an initial correction step. Finally, real-data analysis across multiple preprocessing pipelines confirms that shared-reference transformations can inflate correlation estimates in practice. These results highlight shared-reference preprocessing as a potential source of artificial dependence that should be explicitly considered when interpreting correlation-based findings.

## Introduction

The explosive growth of biological data has led to increasingly complex experimental designs, databases, and analytical frameworks^1,2^. This expansion has created a pressing need for methods that can manage complexity while preserving the validity of downstream inference across both traditional and high-throughput assays. From ELISA and qPCR measurements to large-scale genomic, proteomic, and microbiome datasets, correlation-based inference has become a cornerstone for identifying biological relationships and regulatory interactions. However, the robustness of these analyses can also depend on specific preprocessing choices made before correlation analysis. In particular, when multiple variables are transformed using a shared reference, structural dependencies can emerge that inflate correlation coefficients and distort biological interpretation. Recognizing and addressing these hidden dependencies is essential to ensure that preprocessing clarifies rather than confounds biological inference.

Many preprocessing strategies are used to standardize values across scales, reduce technical variation, and enable fair comparisons between datasets^3^. Some of these strategies involve centering or scaling multiple variables with respect to a common reference or denominator. In those settings, even subtle differences in how the reference is defined can alter correlation patterns and introduce spurious associations^4,5^. Our concern here is not normalization in general, but this specific class of shared-reference transformations and their downstream impact on correlation-based inference.

Despite decades of statistical awareness about ratio-induced dependencies, these issues remain underappreciated in contemporary omics research. Modern high-dimensional datasets magnify the consequences of such artifacts: large-scale correlation matrices are routinely interpreted as biological networks, yet are built on normalized quantities. Unlike classical analyses, these high-throughput contexts amplify small structural biases into global network distortions. Our contribution lies in quantifying this distortion systematically and demonstrating its proportional dependence on the variance of the reference variable (*Z*), a relationship that, to our knowledge, has not been explicitly formalized or illustrated in this way.

Here, we highlight how shared-reference transformations can inadvertently bias correlation analysis, illustrate these artifacts through simulations, and discuss how partial correlation analysis provides a more robust alternative. By drawing attention to these methodological pitfalls, our goal is to encourage a more critical evaluation of preprocessing choices in high-dimensional biological research. Importantly, we study a mechanism that is distinct from standard RNA-seq library-size (sequencing-depth) normalization. Our focus is on downstream shared-reference transformations, where multiple variables are centered or scaled using the same reference, for example, subtracting a common control gene, computing deviations from a shared control group, or dividing by a shared denominator. We show that these steps can introduce additional dependence by construction, even when earlier preprocessing has already reduced depth-related correlation.

## Results

### In silico demonstration: shared references create artificial dependence after normalization

**Fig. 1:**
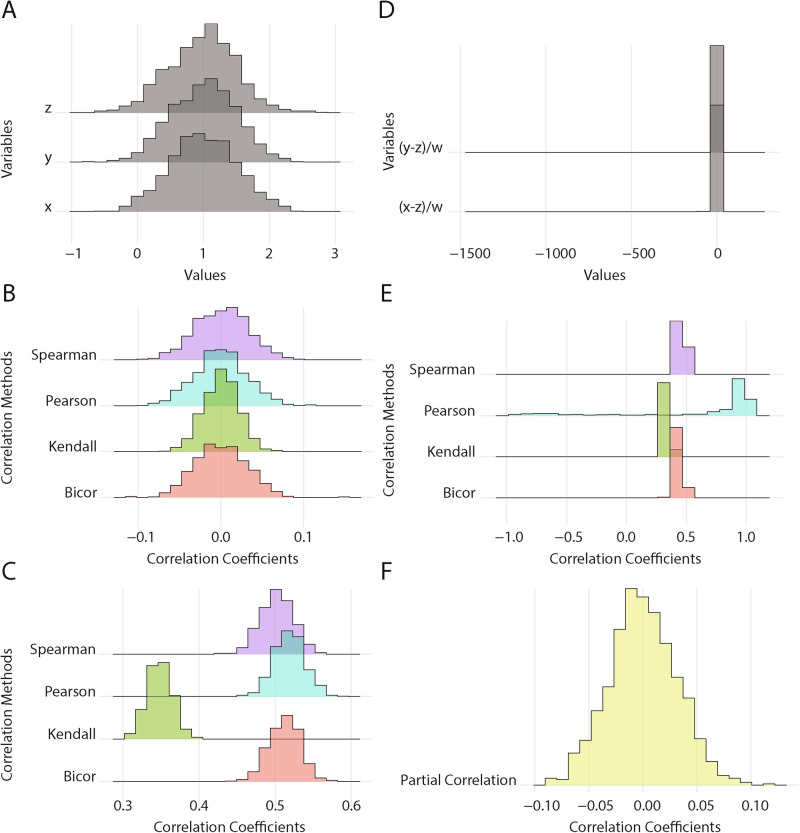
Simulated correlations between independent variables. **A** Distribution of *X*, *Y*, and *Z*. **B** Correlations between *X* and *Y* under Pearson (blue), Spearman (purple), Kendall (green), and bicor (red) tests, all tending towards zero. **C** Correlations between *X*–*Z* and *Y*–*Z*, revealing spurious associations introduced by normalization. **D** Distribution of (*X* –*Z*)/*W* and (*Y*–*Z*)/*W*. **E** Correlation between transformed variables, showing inflated associations. **F** Partial correlation controlling for *Z* and *W* (yellow) removes the artifact, returning correlations to near zero. Simulations used the native rnorm()function in R; histograms generated with ggplot2. See the subsection of the “Methods” section, “Codes” for details.

Here, we analyze shared-reference transformations, in which a common normalizer is reused across variables prior to correlation analysis. This is conceptually different from standard RNA-seq library-size normalization; the key issue is that reusing the same reference term embeds shared stochastic components into multiple variables. To illustrate how shared references can manufacture dependence, we simulated three mutually independent variables (*X*, *Y*, and *Z*) from normal distributions across 1000 individuals (mean = 1, SD = 0.5; Fig. 1A). Under this setting, correlation estimates between *X* and *Y* were centered near zero across Pearson, Spearman, Kendall, and biweight midcorrelation (bicor), consistent with statistical independence (Fig. 1B). Next, we applied a common normalization by subtracting the same reference *Z* from both variables (*X*’ = *X*−*Z* and *Y*’ = *Y*−*Z*). After this transformation, correlation coefficients shifted from near zero to moderate positive values, typically clustering around ~0.5 across methods (Fig. 1C). We then introduced a second independent reference *W* (mean = 1, SD = 0.5) and applied a sequential normalization to the centered variables, computing (*X*−*Z*)/*W* and (*Y*−*Z*)/*W* (Fig. 1D). In this scenario, correlation estimates became strongly distorted, with correlations often approaching extreme values (near ±1), particularly for Pearson’s *r* (Fig. 1E). Finally, when partial correlation was used to condition on the shared reference variable(s), the induced association was attenuated and correlations moved back toward zero (Fig. 1F).

### Systematic sensitivity analysis: normalization artifacts scale with variance and sample size

**Fig. 2:**
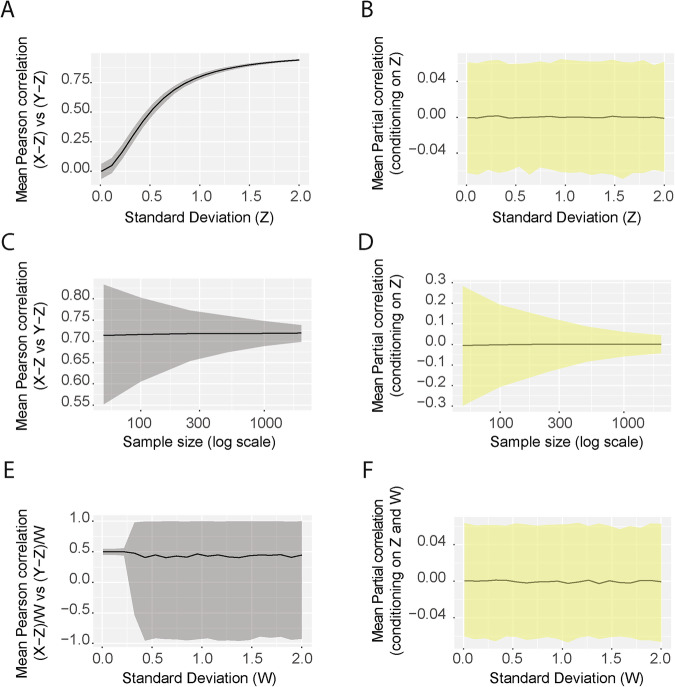
Expanded simulations quantifying normalization-induced artifacts. Effect of the variance of *Z* on correlations between (*X*–*Z*) and (*Y*–*Z*). Higher SD (*Z*) amplifies spurious Pearson correlations (**A**), while partial correlations conditioned on *Z* remain centered near zero (**B**). Effect of sample size (*n*). Larger *n* narrows the confidence intervals but does not eliminate the bias: the mean Pearson correlation remains systematically inflated (**C**), whereas partial correlations stay close to zero (**D**). Sequential normalization scenario. As SD (*W*) increases, Pearson correlations between (*X*–*Z*)/*W* and (*Y*–*Z*)/*W* approach ±1 due to the emergence of heavy tails (Cauchy-like under idealized assumptions) (**E**). Partial correlations conditioned on both *Z* and *W* effectively mitigate the artifact, though instability may persist when *W* approaches zero (**F**). Simulations used rnorm()in R; histograms generated with ggplot2. See the “Methods” subsection “Codes” for details.

To generalize the proof-of-concept, we performed a sensitivity analysis in which we systematically varied key parameters of the normalization procedure, focusing on the dispersion of the shared reference (*Z*), sample size (*n*), and the dispersion of the denominator in a sequential normalization (*W*) (Fig. 2). When *X* and *Y* were simulated as independent variables, increasing the standard deviation of *Z* monotonically increased the mean Pearson correlation between (*X*−*Z*) and (*Y*−*Z*), yielding moderate to strong positive correlations even in the absence of any true association (Fig. 2A). In contrast, partial correlation conditioning on *Z* remained centered near zero across the full range of *Z* variance (Fig. 2B). Next, we assessed whether larger sample sizes mitigate the induced dependence. As n increased, the mean Pearson correlation for the shared subtraction scenario remained essentially unchanged, while uncertainty bands narrowed, indicating that larger cohorts stabilize the spurious correlation rather than removing it (Fig. 2C). Partial correlation conditioning on *Z* remained close to zero and became more stable with increasing n (Fig. 2D). Finally, in the sequential normalization scenario, correlation estimates for (*X*−*Z*)/*W* versus (*Y*−*Z*)/*W* showed marked instability as the standard deviation of *W* varied, with estimates spanning extreme values in some settings (Fig. 2E). Conditioning on both *Z* and *W* reduced the apparent association and brought the mean partial correlation back toward zero (Fig. 2F).

### Illustrative count-based simulation: downstream shared-reference steps can reintroduce dependence after sample-level scaling

**Fig. 3:**
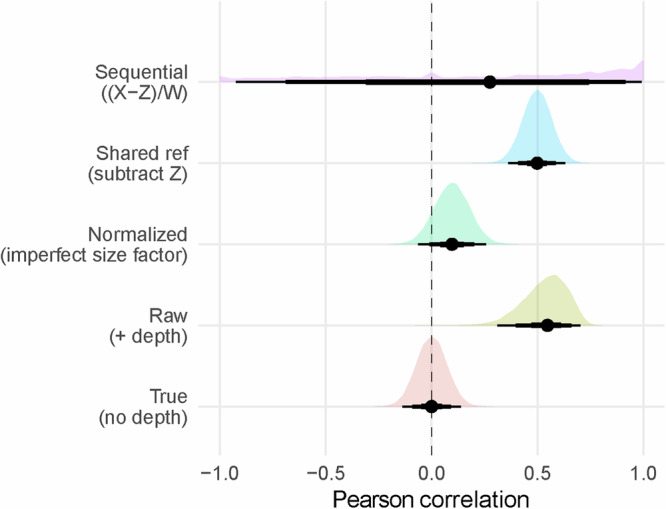
Illustrative count-based simulation: downstream shared-reference steps can reintroduce dependence after sample-level scaling. We simulated a count matrix in which genes are independent at the latent expression level and sample-specific scaling factors induce global dependence in observed counts. Pairwise correlations (Pearson’s *r*) were computed for random gene pairs under five conditions: (i) true latent expression, (ii) raw log-transformed counts after sample-specific scaling, (iii) normalized data after scaling correction using an estimated size factor, (iv) shared-reference transformation after subtracting a common reference gene *Z* from all genes, and (v) sequential shared normalization after applying (*X*−*Z*)/*W* with a second gene *W* as denominator. Distributions are shown as half-violin densities; thick horizontal bars denote the central 50% interval, medium bars the central 80% interval, thin bars the central 95% interval, and points indicate the median. The dashed vertical line marks *r* = 0. The inset illustrates the interval encoding for one representative condition.

In an illustrative count-based simulation with sample-level scaling, the “true” expression matrix before scaling produced correlations centered near zero (mean *r* ≈ 0.0001; SD ≈ 0.071). After applying sample-specific scaling factors, log-transformed raw counts exhibited strong positive correlations among random gene pairs (mean *r* ≈ 0.54; SD ≈ 0.10). After scaling correction using an estimated size factor, correlations in the normalized matrix were reduced relative to the raw counts, although not eliminated (mean *r* ≈ 0.10; SD ≈ 0.08). Introducing a downstream shared-reference transformation then increased dependence again: subtracting a common reference gene *Z* shifted correlations upward (mean *r* ≈ 0.50; SD ≈ 0.07). Sequential shared normalization of the form ((*X*−*Z*)/*W*) produced a more heterogeneous pattern, with a modestly positive mean correlation (mean *r* ≈ 0.19) but a strongly increased dispersion (SD ≈ 0.60), consistent with the instability characteristic of ratio-based transformations (Fig. 3). This simulation is intended as an illustrative count-based example rather than as a benchmark of RNA-seq normalization methods.

### Real-data validation: shared-reference normalization inflates gene–gene correlations across RNA-seq pipelines

To evaluate whether shared-reference artifacts also emerge in real datasets, we performed a randomized resampling analysis on an RNA-seq expression matrix under multiple preprocessing pipelines. In each iteration, we randomly selected one gene (*Z*) to serve as a shared reference and a second gene (*W*) as an additional normalizer. We then computed pairwise correlations among 1000 randomly chosen genes before and after applying the shared-reference transformations, and summarized the effect as the mean correlation and its standard deviation across iterations. To test robustness, we did not fix random seeds between runs. Across several RNA-seq preprocessing pipelines (Raw−log1p counts; no library-size normalization, DESeq2-normalized, edgeR/TMM logCPM, and limma-voom), correlations among random gene pairs were comparatively low in the unmodified matrix for each pipeline, providing a baseline for assessing additional dependence introduced downstream. In contrast, subtracting a common reference (*X*′ = *X*−*Z*; *Y*′ = *Y*−*Z*) systematically shifted the correlation distribution toward positive values, indicating inflation of the correlation coefficient. A similar, and in some settings more variable, inflation was observed after sequential normalization of the form (*X*−*Z*)/*W* and (*Y*−*Z*)/*W*. The corresponding R script is provided in the “Methods” section. Together, these results confirm that shared-reference normalization can generate artificial dependencies in real biological data, and that this artifact persists across several RNA-seq preprocessing frameworks (Table 1).

**Table 1:** Shared-reference transformations inflate gene−gene correlations across RNA-seq normalization pipelines

Transformation	Raw (log1p counts; no library-size normalization) Mean	SD	*n* (iterations)	DESeq2 Mean	SD	*n* (iterations)	edgeR (TMM+ logCPM) Mean	SD	*n* (iterations)	limma-voom Mean	SD	*n* (iterations)
No shared reference (original)	0.02	0.24	500	0.01	0.23	500	0.06	0.30	500	0.20	0.40	500
Shared subtraction (*X*−*Z*, *Y*−*Z*)	0.59	0.46	500	0.58	0.25	500	0.72	0.19	500	0.62	0.19	500
Sequential normalization ((*X*−*Z*)/*W*, (*Y*−*Z*)/*W*)	0.36	0.81	500	0.56	0.25	500	0.54	0.29	500	0.55	0.28	500

For each iteration (*n* = 500), one gene was randomly selected as a shared reference (*Z*) and another as a secondary normalizer (*W*). We then sampled 1000 genes and computed pairwise correlations among random gene pairs under each preprocessing pipeline, before and after applying shared-reference subtraction (*X*′ = *X*−*Z*; *Y*′ = *Y*−*Z*) and sequential shared normalization ((*X*−*Z*)/*W*, (*Y*−*Z*)/*W*). Reported values are the mean and standard deviation of correlation coefficients across iterations; random seeds were not fixed. Correlation metric: Pearson’s *r*.

## Discussion

Shared-reference transformations can generate structured dependence in correlation-based analyses. When multiple variables are centered or scaled using the same reference or denominator, the resulting quantities inherit shared stochastic components that can be mistaken for biological association. Across the analytical derivation (see the “Methods” section), controlled simulations (Figs. 1 and 2), an illustrative count-based example (Fig. 3), and a real RNA-seq case study (Table 1), our results show that this effect is systematic, increases with reference variability, and is not resolved simply by increasing sample size. Partial correlation can attenuate the artifact when the relevant reference variables are available, but ratio-based transformations may still introduce instability that complicates interpretation^6^.

The analytical result provides the core intuition. If *X*, *Y*, and *Z* are mutually independent, subtracting the same reference *Z* from both *X* and *Y* induces a nonzero expected correlation between (*X*−*Z*) and (*Y*−*Z*). In particular, normalization by a shared reference *Z* induces a correlation equal to

1 $$P(X-Z),(Y-Z)=\,\frac{\mathrm{Var}(Z)}{\sqrt{(\mathrm{Var}(X)+\,\mathrm{Var}(Z))(\mathrm{Var}(Y)+\mathrm{Var}(Z))}}$$

which approaches 1 as Var(*Z*) becomes dominant (see the “Methods” section). This follows because:

2 $$\mathrm{Cov}(X-Z,\,Y-Z)=\mathrm{Var}(Z)$$

The key point is that the inflated correlation does not reflect a genuine association between *X* and *Y*, but a shared dependence on *Z*. This is especially relevant because correlation analysis is widely used to infer biological coupling and co-regulation in bioinformatics and systems biology^7,8^. Even when the coefficient is computed correctly, its interpretation can be distorted if the data have been structured by shared-reference preprocessing rather than by the underlying biology.

Changing the correlation metric does not solve this problem. Rank-based and robust estimators can reduce sensitivity to outliers and deviations from normality^9–11^, but they do not remove dependence introduced earlier by shared-reference preprocessing. A robust estimator may be less influenced by extreme values, yet still quantify an association that was created during preprocessing.

Our simulations also clarify how these effects can emerge in specific analytical workflows, including sequential preprocessing. When the same or related references are reused across multiple steps, shared components can accumulate and become embedded in the final variables used for correlation analysis. In our toy examples, *z*-score transformation is generally well behaved when the reference is stable (Fig. 1D), whereas group-referenced deviation metrics such as “molecular distance from healthy” can become problematic if they are later repurposed for correlation-based inference^12^. Although such metrics were originally introduced as dissimilarity scores, they have also been used in tasks such as outlier detection^13^ and harmonization or batch correction in integrative studies^14^, contexts in which downstream correlation-based interpretation may be attempted.

Ratio-based steps deserve particular attention because they can amplify noise when denominators are volatile or near zero. Under idealized assumptions, the transformation (*X*−*Z*)/*W* can exhibit heavy-tailed behavior and produce extreme realizations^15,16^. In practice, the exact distribution may vary depending on biological and preprocessing constraints, but the central issue remains numerical instability, especially as *W* approaches zero. This helps explain why sequential shared normalization can generate highly dispersed and sometimes extreme correlation estimates, even when the underlying variables are not strongly associated.

The real-data case study reinforces that these mechanisms are not merely theoretical. Across several RNA-seq preprocessing pipelines (Table 1), the same dataset yielded materially different correlation patterns after shared-reference transformations were applied, indicating that some inferred gene-gene associations can be contingent on preprocessing rather than biologically intrinsic. Although the observed shifts were smaller and more heterogeneous than in the idealized simulations, they followed the same general pattern: earlier preprocessing may reduce depth-related dependence, but additional shared-reference transformations applied downstream can reintroduce artificial structure into the data.

From a broader perspective, estimating direct associations after adjusting for confounders is a natural extension of network analysis in systems biology^17,18^, particularly as high-throughput datasets continue to expand across genomics, transcriptomics, proteomics, and metabolomics^19^. In this context, partial correlation is a useful but limited corrective. Its effectiveness depends on linearity assumptions and on the availability and reliability of the variables used for adjustment. In practice, reference quantities are often estimated rather than directly observed, and their uncertainty can propagate through the correction step. In compositional datasets, the constant-sum constraint further limits standard correlation and partial correlation, making log-ratio approaches within the Aitchison framework more appropriate^20^.

Taken together, our results show that shared-reference preprocessing should be treated as a methodological choice with direct consequences for inference, not as a neutral analytical step. When shared references or denominators are used, their stability should be assessed explicitly, and correlation-based findings should be interpreted in light of how the input variables were constructed. Recognizing these predictable artifacts as structured biases can improve the reproducibility and interpretability of correlation-based analyses in omics and related high-dimensional biological data.

## Methods

### Data simulation

All simulations were performed in R version 4.2.2 (October 31, 2022). Random variables were generated using rnorm(), simulating 1000 individuals with 1000 resampling iterations.

### Real data

We analyzed transcriptomic profiles from the dataset PRJEB9292^21^. This dataset comprises expression values from human bronchial epithelial cells (normal and cystic fibrosis donors) exposed to *Pseudomonas aeruginosa*, quantified through RNA-seq.

### Correlation analysis

Pairwise correlations were calculated using the cor() function with Pearson, Spearman, and Kendall methods. Biweight midcorrelation (bicor) was computed with the bicor() function from the WGCNA package^11^. Partial correlations were estimated using the ppcor package^18^.

### Visualization

Density plots and histograms were generated using the ggplot2 package and native R plotting functions.

### Codes

## The following code snippets reproduce the simulations presented in Figs. 1–3.

## Required packages

library(ppcor) # partial correlation

library(WGCNA) # bicor

library(ggplot2)

# -----------------------------

# Global parameters

# -----------------------------

n <- 1000 # number of individuals (samples)

resampling <- 1000 # number of Monte Carlo iterations

# Helper: compute multiple correlations

compute_corrs <- function(x, y) {

c(

pearson = cor(x, y, method = “pearson”),

spearman = cor(x, y, method = “spearman”),

kendall = cor(x, y, method = “kendall”),

bicor = bicor(x, y)

)

}

# -----------------------------

# 1) Fig. 1B: baseline correlations (X, Y independent)

# -----------------------------

cors_baseline <- matrix(NA, nrow = resampling, ncol = 4)

colnames(cors_baseline) <- c(“pearson”,“spearman”,“kendall”,“bicor”)

for (i in 1:resampling) {

x <- rnorm(n, mean = 1, sd = 0.5)

y <- rnorm(n, mean = 1, sd = 0.5)

cors_baseline[i,] <- compute_corrs(x, y)

}

# Example plot

plot(density(cors_baseline[, “pearson”]), main = “Baseline (Pearson)”, xlab = “*r*”)

# -----------------------------

# 2) Fig. 1C and F: shared-reference subtraction (Z) and partial correlation

# -----------------------------

cors_sub <- numeric(resampling)

pcor_sub <- numeric(resampling)

z <- rnorm(n, mean = 1, sd = 0.5)

for (i in 1:resampling) {

x <- rnorm(n, mean = 1, sd = 0.5)

y <- rnorm(n, mean = 1, sd = 0.5)

cors_sub[i] <- cor(x - z, y - z, method = “pearson”)

pcor_sub[i] <- pcor.test(x, y, z)$estimate

}

hist(cors_sub, main = “Shared subtraction: cor(X−Z, Y−Z)”, xlab = “r”)

hist(pcor_sub, main = “Partial correlation conditioning on Z”, xlab = “partial *r*”)

# -----------------------------

# 3) Fig. 1E and F: sequential shared normalization ((X−Z)/W) and partial correlation

# -----------------------------

cors_seq <- numeric(resampling)

pcor_seq <- numeric(resampling)

w <- rnorm(n, mean = 1, sd = 0.5)

for (i in 1:resampling) {

x <- rnorm(n, mean = 1, sd = 0.5)

y <- rnorm(n, mean = 1, sd = 0.5)

cors_seq[i] <- cor((x−z)/w, (y−z)/w, method = “pearson”)

pcor_seq[i] <- pcor.test(x, y, cbind(z, w))$estimate

}

hist(cors_seq, main = “Sequential: cor((X−Z)/W, (Y−Z)/W)”, xlab = “r”)

hist(pcor_seq, main = “Partial correlation conditioning on Z and W”, xlab = “partial r”)

# -----------------------------

# 4) Analytical approximation used in the text

# cor(X−Z, Y−Z) = var(Z)/sqrt((var(X) + var(Z))*(var(Y) + var(Z)))

# under independence between X, Y and Z

# -----------------------------

cor_shared_ref<-function(var_x, var_y, var_z) {

var_z/sqrt((var_x + var_z) * (var_y+; var_z))

}

# Example curve as var(Z) changes

var_x <- 0.5^2

var_y <- 0.5^2

var_z_grid <- seq(0.01, 9, length.out = 200)

curve_vals <- sapply(var_z_grid, function(vz) cor_shared_ref(var_x, var_y, vz))

plot(var_z_grid, curve_vals, type = “l”,

xlab = “var(Z)”, ylab = “Expected cor(X-Z, Y-Z)”)

# -----------------------------

# 5) Fig. 2: parameter sweep (variance of Z, sample size n, variance of W)

# -----------------------------

# A) vary sd(Z)

sd_z_values <- seq(0.1, 3, by = 0.1)

mean_r_sdZ <- numeric(length(sd_z_values))

mean_pcor_sdZ <- numeric(length(sd_z_values))

for (k in seq_along(sd_z_values)) {

sd_z <- sd_z_values[k]

z <- rnorm(n, mean = 1, sd = sd_z)

r_vals <- numeric(resampling)

p_vals <- numeric(resampling)

for (i in 1:resampling) {

x <- rnorm(n, mean = 1, sd = 0.5)

y <- rnorm(n, mean = 1, sd = 0.5)

r_vals[i] <- cor(x - z, y - z)

p_vals[i] <- pcor.test(x, y, z)$estimate

}

mean_r_sdZ[k] <- mean(r_vals)

mean_pcor_sdZ[k] <- mean(p_vals)

}

df_sdZ <- data.frame(sdZ = sd_z_values, mean_r = mean_r_sdZ, mean_pcor = mean_pcor_sdZ)

plot(df_sdZ$sdZ, df_sdZ$mean_r, type = “l”, xlab = “sd(Z)”, ylab = “Mean Pearson r”)

# B) vary sample size n

n_values <- c(50, 100, 200, 500, 1000, 2000)

mean_r_n <- numeric(length(n_values))

mean_pcor_n <- numeric(length(n_values))

for (k in seq_along(n_values)) {

n_k <- n_values[k]

z <- rnorm(n_k, mean = 1, sd = 0.5)

r_vals <- numeric(resampling)

p_vals <- numeric(resampling)

for (i in 1:resampling) {

x <- rnorm(n_k, mean = 1, sd = 0.5)

y <- rnorm(n_k, mean = 1, sd = 0.5)

r_vals[i] <- cor(x - z, y - z)

p_vals[i] <- pcor.test(x, y, z)$estimate

}

mean_r_n[k] <- mean(r_vals)

mean_pcor_n[k] <- mean(p_vals)

}

df_n <- data.frame(n = n_values, mean_r = mean_r_n, mean_pcor = mean_pcor_n)

plot(df_n$n, df_n$mean_r, type = “b”, xlab = “n”, ylab = “Mean Pearson r”)

# C) vary sd(W) in sequential normalization

sd_w_values <- seq(0.1, 3, by = 0.1)

mean_r_sdW <- numeric(length(sd_w_values))

mean_pcor_sdW <- numeric(length(sd_w_values))

z <- rnorm(n, mean = 1, sd = 0.5)

for (k in seq_along(sd_w_values)) {

sd_w <- sd_w_values[k]

w <- rnorm(n, mean = 1, sd = sd_w)

r_vals <- numeric(resampling)

p_vals <- numeric(resampling)

for (i in 1:resampling) {

x <- rnorm(n, mean = 1, sd = 0.5)

y <- rnorm(n, mean = 1, sd = 0.5)

r_vals[i] <- cor((x - z) / w, (y - z) / w)

p_vals[i] <- pcor.test(x, y, cbind(z, w))$estimate

}

mean_r_sdW[k] <- mean(r_vals)

mean_pcor_sdW[k] <- mean(p_vals)

}

df_sdW <- data.frame(sdW = sd_w_values, mean_r = mean_r_sdW, mean_pcor = mean_pcor_sdW)

plot(df_sdW$sdW, df_sdW$mean_r, type = “l”, xlab = “sd(W)”, ylab = “Mean Pearson r”)

# -----------------------------

# 6) Fig. 3: RNA-seq-like simulation with library size + imperfect normalization

# -----------------------------

G < - 5000

n <- 200

theta <- 20

sigma_s <- 0.7

sigma_eta <- 0.2

mu_alpha <- 2

sd_alpha <- 1

sigma_eps <- 0.3

alpha_g <- rnorm(G, mu_alpha, sd_alpha)

eps_gi <- matrix(rnorm(G * n, 0, sigma_eps), nrow = G, ncol = n)

log_mu <- alpha_g + eps_gi

mu <- exp(log_mu)

# Library size (depth)

s <- exp(rnorm(n, 0, sigma_s))

# Raw counts with depth (NB)

C <- matrix(

rnbinom(G * n, size = theta, mu = as.vector(mu * rep(s, each = G))),

nrow = G, ncol = n

)

# Imperfect size-factor estimate

s_hat <- exp(log(s) + rnorm(n, 0, sigma_eta))

# Normalized expression

N <- log1p(sweep(C, 2, s_hat, “/“))

# Shared reference downstream

Z <- sample(1:G, 1)

W <- sample(setdiff(1:G, Z), 1)

N_sub <- sweep(N, 2, N[Z,], “-“)

N_seq <- sweep(N_sub, 2, pmax(N[W,], 1e-6), “/“)

# Sample random gene pairs and compute correlations

sample_pair_cor <- function(M, n_genes = 1000, n_pairs = 50000, method = “pearson”) {

g_idx <- sample(1:nrow(M), n_genes)

a <- sample(g_idx, n_pairs, replace = TRUE)

b <- sample(g_idx, n_pairs, replace = TRUE)

same <- a == b

while (any(same)) {

b[same] <- sample(g_idx, sum(same), replace = TRUE)

same <- a == b

}

mapply(function(i, j) cor(M[i,], M[j,], method = method), a, b)

}

cor_true <- sample_pair_cor(log1p(mu))

cor_raw <- sample_pair_cor(log1p(C))

cor_norm <- sample_pair_cor(N)

cor_sub2 <- sample_pair_cor(N_sub)

cor_seq2 <- sample_pair_cor(N_seq)

summary_stats <- function(x) c(mean = mean(x), sd = sd(x))

rbind(

true = summary_stats(cor_true),

raw = summary_stats(cor_raw),

norm = summary_stats(cor_norm),

sub = summary_stats(cor_sub2),

seq = summary_stats(cor_seq2)

)

## Supplementary information

Supplementary Information

## Data Availability

The RNA-seq dataset analyzed in this study is publicly available from the European Nucleotide Archive under accession **PRJEB9292** .

## Code availability

Code is provided in the “Methods” section.
